# Biodiversity of *Photobacterium* spp. Isolated From Meats

**DOI:** 10.3389/fmicb.2019.02399

**Published:** 2019-10-18

**Authors:** Sandra Fuertes-Perez, Philippa Hauschild, Maik Hilgarth, Rudi F. Vogel

**Affiliations:** Lehrstuhl Technische Mikrobiologie, Technische Universität München, Freising, Germany

**Keywords:** *Photobacterium carnosum*, *Photobacterium phosphoreum*, *Photobacterium iliopiscarium*, meat spoilage, psychrophilic spoilers, modified atmosphere packaging

## Abstract

Photobacteria are common psychrophilic bacteria found in marine environments. Recently, several studies revealed high numbers of *Photobacterium* (*P.*) spp. on packaged fresh meat. Their occurrence appears relevant for the spoilage of meat, since species of the genus are already known as potent fish spoilage organisms. Here we report on distribution, biodiversity, and specific traits of *P. carnosum* (*n* = 31), *P. phosphoreum* (*n* = 24), and *P. iliopiscarium* (*n* = 3) strains from different foods. Biodiversity was assessed by genomic fingerprinting, diversity index analysis, growth dynamics, comparison of metabolic activities, and antibiotic resistance. We observed a ubiquitous occurrence of the species on all common meats independent of packaging conditions and producer, suggesting contamination during an established processing or packaging step. Regarding biodiversity, the three species differed clearly in their growth properties and metabolic characteristics, with *P. phosphoreum* growing the fastest and showing the strongest alkalization of the media. On strain level we also recorded variations in enzymatic reactions, acid production, and antibiotic resistances not restricted to specific meat types. This depicts high biodiversity on species and strain level on each contaminated meat sample. Our analysis showed that meat-borne strains of *P. phosphoreum* and *P. iliopiscarium* clearly differ from their type strains from a marine habitat. Additionally, we report for the first time isolation of *P. carnosum* strains from packaged fish, which in contrast showed comparable phenotypic properties to meat-borne strains. This hints at different initial origins of *P. phosphoreum/P. iliopiscarium* (marine background) and *P. carnosum* (no demonstrated marine background) contaminations on fish and meat, respectively.

## Introduction

Photobacteria are Gram-negative, facultatively aerobic members of the Vibrionaceae family and well known as marine-related species (Lo et al., 2014; Li et al., 2017; Wang et al., 2017). First described in 1889 (Beijerinck, 1889), the genus currently comprises 30 valid species, and 2 subspecies (Parte, 2018). Photobacteria occur free-living in seawater and sediments or in interaction with marine animals (Urbanczyk et al., 2011; Labella et al., 2017), e.g., the symbiosis of bioluminescent strains within the light organs of deep sea fish (Hendrie et al., 1970). However, photobacteria are also known as effective saprotrophs in marine habitats (Urbanczyk et al., 2011). In this context, certain species, i.e., *Photobacterium* (*P.*) *phosphoreum* and *P. iliopiscarium* constitute a considerable problem in the food industry, representing potent spoilers of chilled fish and seafood products (Okuzumi et al., 1994; Dalgaard et al., 1997). The spoilage processes involve production of biogenic amines such as histamine (Okuzumi et al., 1994; Jorgensen et al., 2000; Emborg et al., 2002; Torido et al., 2012; Takahashi et al., 2015; Bjornsdottir-Butler et al., 2018) that can lead to scombroid fish poisoning (Lehane and Olley, 2000).

Previous studies based on culture-independent approaches have revealed presence of photobacteria gene sequences on pork (Nieminen et al., 2016), pork sausages (Bouju-Albert et al., 2018), beef (Pennacchia et al., 2011), and minced meat (Stoops et al., 2015; Nieminen et al., 2016). In only one of these studies very few isolates of *P. phosphoreum* were recovered (Nieminen et al., 2016) since common control methods rely on standard agars and cultivation at temperatures between 25 and 30°C, which do not allow isolation of fastidious and psychrophilic photobacteria. Highly frequent isolation was recently demonstrated by Hilgarth et al. (2018a) employing a novel, targeted selective isolation procedure for recovery of photobacteria from foods. *P. phosphoreum* and *P. iliopiscarium* were isolated from modified atmosphere packaged (MAP) poultry, pork, and beef (only *P. phosphoreum*) (Hilgarth et al., 2018a). *P. phosphoreum* was firstly described in 1878 (Cohn, 1878) and re-evaluated in 1889 (Beijerinck, 1889) as a luminous isolate from the sea. It is adapted to high-pressure (Labella et al., 2017), grows optimally at 15–20°C, and occurs frequently as predominant spoiler on fish products (Gram and Huss, 1996; Reynisson et al., 2009). *P. iliopiscarium* was described by Onarheim et al. (1994) as *Vibrio iliopiscarium* and later reassigned to *Photobacterium* by Urakawa et al. (1999). There are several studies reporting *P. iliopiscarium* isolates from marine fish (Dunlap and Ast, 2005; Olofsson et al., 2007; Thyssen and Ollevier, 2015; Hilgarth et al., 2018a) but only few that describe them as predominant (Olofsson et al., 2007). Just as *P. phosphoreum*, it prefers 15–20°C for growth (Onarheim et al., 1994; Hilgarth et al., 2018b). In addition, a new psychrophilic species, *P. carnosum*, was recently discovered on meat. It also prefers 10–15°C and was described as the first species of the genus that is unrelated to marine habitats (Hilgarth et al., 2018b). This new species was reported as the major representative of the *Photobacterium* genus on poultry and beef, while it was less abundant on pork.

Not only do these psychrophilic bacteria occur in high numbers on meat, but they also exhibit spoilage potential. A recent metatranscriptomic study has predicted its potential for production of several biogenic amines, such as putrescine, cadaverine, agmatine, tyramine, and gamma-amino-butyric acid as well as various other spoilage compounds that are known for other potent meat spoilers (Höll et al., 2019).

Until now, knowledge on the origin and biodiversity of *P. carnosum*, *P. phosphoreum*, and *P. iliopiscarium* on food products and especially meats is very limited. This study aimed at elucidation of their distribution and diversity in order to identify specific traits of the species and possible correlations between the source of isolation, genotypes, or physiotypes. For this, we surveyed and reviewed the occurrence of photobacteria on meat samples from local butchers and supermarkets. Selected isolates from different samples were then used to thoroughly study biodiversity.

## Materials and Methods

### Isolation and Identification of Photobacteria

Isolation was carried out as described in the isolation protocol from Hilgarth et al. (2018a). Samples purchased and kept at 4°C were cut and homogenized in marine broth (DIFCO). Samples were plated on marine agar [marine broth, 1.6% agar-agar (w/v)] supplemented with 3 g/L meat extract and 7 mg/L vancomycin, and incubated at 15°C for 72 h. Composition of the base marine broth media includes: peptone 5 g/L, yeast extract 1 g/L, sodium chloride 19.45 g/L, ferric citrate 0.1 g/L, magnesium chloride 5.9 g/L, magnesium sulfate 3.24 g/L, calcium chloride 1.8 g/L, potassium chloride 0.55 g/L, sodium bicarbonate 0.16 g/L, potassium bromide 0.08 g/L, strontium chloride 34 mg/L, boric acid 22 mg/L, sodium silicate 4 mg/L, sodium fluoride 2.4 mg/L, ammonium nitrate 1.6 mg/L, and disodium phosphate 8 mg/L. Isolates were identified based on their low-molecular subproteome with MALDI-TOF MS on a Microflex LT Spectrometer (Bruker Corporation, Billerica, MA, United States) by direct transfer method and on-target extraction (Usbeck et al., 2013; Hilgarth et al., 2018a). An in-house database containing mass spectrometry profiles of various photobacteria species was established by sequencing of housekeeping genes in order to guarantee reliable identification. In total at least three packages per meat type were analyzed for abundance of photobacteria. Type strains *P. phosphoreum* DSM15556^T^ and *P. iliopiscarium* DSM9896^T^, obtained from the German Strain Collection (DSMZ), were also part of the selected strains. Additionally, the type strain *P. carnosum* TMW2.2021^T^ and some already described strains of the species (TMW2.2022, TMW2.2029, and TMW2.2030) were included (Hilgarth et al., 2018b).

### Genomic Fingerprinting

Randomly amplified polymorphic DNA (RAPD)-PCR fingerprinting was used to assess the number of different strains within all isolates and select them for subsequent characterization. RAPD-PCR was performed with the primer M13V (5′-GTT TTC CCA GTC ACG AC-3′) (Ehrmann et al., 2003). Bands were separated by electrophoresis in agarose gel (1.4% w/v, 150 V, 2.5 h). Lambda DNA/*Eco*RI plus *Hin*dIII Marker (Thermo Scientific, Hampshire, United Kingdom) was used as molecular weight marker and for normalization/standardization of the gel pattern for comparison. Similarities in fingerprint pattern were analyzed with Bionumerics V7.6.2 (Applied Maths, Sint-Martens-Latem, Belgium). Hierarchical clustering analysis was carried out by unweighted pair group method with arithmetic mean (UPGMA) method and Dice similarity coefficient with 1% tolerance. After the initial strain delineation by RAPD-PCR for all isolates, the RAPD approach was again performed twice for all strains of the three species to assess the reproducibility of the observed patterns and ensure the fidelity of the clustering. Furthermore, the similarity of triplicates of all strains was compared to the triplicates of the closest related strain in order to further validate the strain delineation and distinctness.

Randomly amplified polymorphic DNA PCR protocol was additionally carried out with primer M14V (5′-CTG TCC AGT CAC GTC-3′) with all selected strains in order to confirm their distinctness and diversity within the species. Protocol and standardization was performed as described for M13V primer.

### Diversity Index Analysis

Individual rarefaction analysis and calculation of diversity indices for evenness (Simpson, 1949), entropy (Shannon and Weaver, 1949), and richness (Chao, 1984) was performed using PAST software 3.25 (Hammer et al., 2001) with operational taxonomic units [OTUs (Schloss and Handelsman, 2005)] defined as distinct/unique RAPD genomic fingerprinting representing distinct genotypes on strain level. A *p*-value < 0.05 was defined as significantly different. Coverage (%) of genotypes was calculated using Good’s coverage estimator as described by Good (1953) with the equation:

(1) $$C=\left(1-\frac{N_{1}}{n}\right)*100,$$

with *N*_1_ representing OTUs only found once (singletons) and *n* as the total number of individuals (strains).

### Growth Analysis in Meat Simulation Medium

Growth curves were performed with all isolated strains of the three species of photobacteria used in this study, a total of 31 strains of *P. carnosum*, 24 strains of *P. phosphoreum*, and 3 strains of *P. iliopiscarium*, in addition to the marine type strains of *P. phosphoreum* (DSM15556^T^) and *P. iliopiscarium* (DSM9896^T^). Inoculum was prepared from an overnight culture in marine broth at 15°C, by centrifuging the cells (4000 × *g*, 10 min), washing them with NaCl 2% (w/v), and resuspending on meat-simulation media. Growth curves were started by inoculating meat-simulation media (20 g/L meat extract, 20 g/L NaCl, pH 5.8) in 50 mL Erlenmeyer flasks at an initial OD_600_ of 0.05. Cultures were incubated at 4°C with constant agitation, and samples were taken regularly for OD_600_ measurement. The pH of the culture was measured at maximum OD_600_. Growth curves were adjusted to parametric models with RStudio v1.1.463 and grofit package v.1.1.1-1 (Kahm et al., 2010) to determine lag phase (lag), maximum growth rate (U), and maximum OD_600_. Growth curves were performed in triplicates and data were further analyzed in IBM SPSS Statistics v23.0.0.0. Tests for normality (Shapiro–Wilk) and homogeneity of variances (Levene test) were carried out for each set of data. One-way ANOVA followed by HSD Tukey *post hoc* test determined significant differences between the strains of each species. Welch-ANOVA and Games-Howell *post hoc* tests were used in case of heterogeneity of variances. Significance level was determined by *p* < 0.05.

### Motility Test

Motility for all strains was determined by the soft agar stab method. Meat-simulation media supplemented with 3 g/L agar was poured into tubes. Motility was measured based on the turbidity of the soft agar around the stabbing zone.

### Bioluminescence of *P. phosphoreum* Strains

Bioluminescence in darkness was scored by visual comparison of the intensity on marine agar plates for all *P. phosphoreum* strains. Suspensions with the same OD_600_ were prepared for all strains, plated on marine agar plates, and incubated at 15°C for 72 h.

### Antibiotic Resistance Test

Antibiotic resistance of all strains of the three species of photobacteria was assessed by disc diffusion assay. All discs were purchased from Oxoid (Thermo Scientific, Hampshire, United Kingdom).

### Metabolic Characterization

Metabolic characterization was assessed for a representative group of all the strains of the three species of photobacteria. A total of 14 strains of *P. phosphoreum*, 16 strains of *P. carnosum*, and 3 strains of *P. iliopiscarium* were assessed for carbohydrate acid production and enzymatic activities. Production of acid from different carbon sources was assessed by the API 50CH test (bioMérieux, Marcy-l’Étoile, France). Several enzymatic activities were tested with the API ZYM test (bioMérieux, Marcy-l’Étoile, France) according to the instructions from the manufacturer. Both procedures were performed according to the methodology followed by Hilgarth et al. (2018b) and data for *P. carnosum* TMW2.2021/2.2022/2.2029/2.2030, *P. phosphoreum* DSM15556^T^, and *P. iliopiscarium* DSM9896^T^ were taken from this study.

### Hierarchical Cluster Analysis

Hierarchical cluster based on the results for physiological tests of selected strains was carried out by a Heatmapper tool^1^ with average linkage criteria and Euclidean distance.

## Results

### Occurrence of Photobacteria on Selected Food Products

Various food samples were obtained from local retailers and butchers and screened on the presence of photobacteria. We detected them on several meat types and on marine fish (Table 1), on MAP packaged, vacuum packaged, and air stored samples and also on marinated meats. The contaminated samples originated thereby from large supermarket chains as well as from small local shops. However, not all samples contained photobacteria, even if they originated from the same producer. We also found different species compositions that were dependent on the meat type. In addition to our previously published data, we identified only two species – *P. carnosum* and *P. phosphoreum* – on beef and turkey. On chicken and pork, and additionally on salmon, we detected *P. carnosum*, *P. phosphoreum*, and *P. iliopiscarium* (Table 1). Besides different meats, we analyzed a variety of additional food products to determine the distribution of photobacteria in the food industry. We did not detect photobacteria in algae (dried and salad), ready-to-eat salad (MAP, 2 samples), and sprouts (MAP); raw milk (12 samples), mozzarella cheese (3 samples), and eggs (3 samples); scallops (defrosted), trout, shrimps (cooked, defrosted) and sea salt; and minced meat (beef and mixed, 5 samples), bacon (2 samples), cooked ham, raw ham, and dried meat (pork).

**TABLE 1:** Detection of *Photobacterium* spp. on different meats.

**Packaging**	**Meat type**	**Origin**	**Detected**	**Relative abundance of**	**CFU photobacteria**	**CFU bacteria**
**atmosphere**			***Photobacterium* spp.**	***Photobacterium* spp. (%)**	**[log_10_(CFU/g)]**	**[log_10_(CFU/g)]**
Air	Chicken	Local butchery	*P. carnosum*	100	6.29	7.67
Air	Beef	Local butchery	*P. carnosum*	100	7.54	9.22
Air	Pork	Local butchery	*P. phosphoreum*	100	8.57	9.34
Air	Codfish	Local fish shop	*P. phosphoreum*	100	NA	NA
Air	Marinated turkey	Supermarket	*P. carnosum P. phosphoreum*	25	7.17	8.28
				75		
MAP	Marinated chicken	Supermarket	*P. carnosum P. phosphoreum*	96	4.54	4.63
				4		
MAP	Marinated beef	Supermarket	*P. phosphoreum*	100	8.76	9.66
MAP	Chicken^∗^	Supermarket	*P. carnosum P. phosphoreum P. iliopiscarium*	71	6.56	6.57
				27		
				2		
MAP	Beef^∗^	Supermarket	*P. carnosum P. phosphoreum*	90	3.55	4.19
				9		
MAP	Pork^∗^	Supermarket	*P. carnosum P. phosphoreum P. iliopiscarium*	5	7.07	7.13
				26		
				69		
MAP	Salmon	Supermarket	*P. carnosum P. phosphoreum P. iliopiscarium Photobacterium* sp.	7	6.77	6.8
				58		
				22		
				13		
Vacuum	Beef	Supermarket	*P. carnosum Photobacterium* sp.	96	6.72	6.72
				4		
Vacuum	Pork	Supermarket	*P. carnosum Photobacterium* sp.	99	7.15	7.15
				1		

*Representative types of spoiled meat samples where photobacteria were detected, and the common distribution of photobacteria found on them. The meat samples were bought in different supermarkets and shops and then incubated at 4°C until they were expired. Its spoilage community on selective medium was then analyzed with MALDI-TOF MS. CFU was determined on the base of the selective media consisting on marine broth supplemented with 3 g/L meat extract and 7 mg/L vancomycin. ^∗^Information obtained from Hilgarth et al. (2018a) and appended for comparison. NA, quantification was not possible due to overgrow of bacteria on plates, but photobacteria were recovered by observing bioluminescent colonies.*

### Genetic Differentiation

In total, we recovered 163 *P. carnosum*, 113 *P. phosphoreum*, and 3 *P. iliopiscarium* isolates from chicken, turkey, pork beef, and salmon (total *n* = 279). Based on differences in their RAPD pattern obtained with primer M13V, we were able to discriminate 31 strains of *P. carnosum*, 24 of *P. phosphoreum*, and 3 strains of *P. iliopiscarium* within all isolates for further investigations on biodiversity. Genotypic distinctness of the strains were further validated with a RAPD approach using primer M14V. Isolates of *P. phosphoreum* from MAP farmed salmon showed no distinct or unique genotypes and were therefore considered as redundant strains. However, we recovered two strains of *P. carnosum* from salmon that were not abundant on other meats. Additional detailed information regarding the sample of origin of every strain used in this study can be found in Supplementary Table S1.

Calculation of diversity indices (Table 2) and an individual rarefaction analysis (Supplementary Figure S1) were carried out for all strains of each species with OTUs based on distinct genomic fingerprinting patterns. The analysis demonstrated that biodiversity of *P. phosphoreum* and *P. carnosum* was completely or almost completely covered by the strains isolated in this study, respectively. This was indicated by saturated rarefaction curves, a high calculated coverage value (>99%, >96%) and an identical or very similar richness of the expressed Chao-1 value to the actual number of genotypes. Additionally, both species were not significantly different regarding their ecological evenness and entropy (*p* > 0.05). Regarding *P. iliopiscarium*, calculation of diversity indices and comparison to the other two species were not expedient since only three isolates with three different genotypes could be recovered.

**TABLE 2:** Diversity indices of photobacteria species using genotyping OTUs.

**Species**	***P. phosphoreum***	***P. carnosum***	***P. iliopiscarium***
Individuals (isolates)	113	163	3
OTUs (strains)	24	31	3
Simpson (evenness)	0.9526	0.9406	–
Shannon (entropy)	3.106	3.081	–
Chao-1 (richness)	24	34.75	–
Good’s coverage estimator (%)	99.12	96.32	0

**FIGURE 1:**
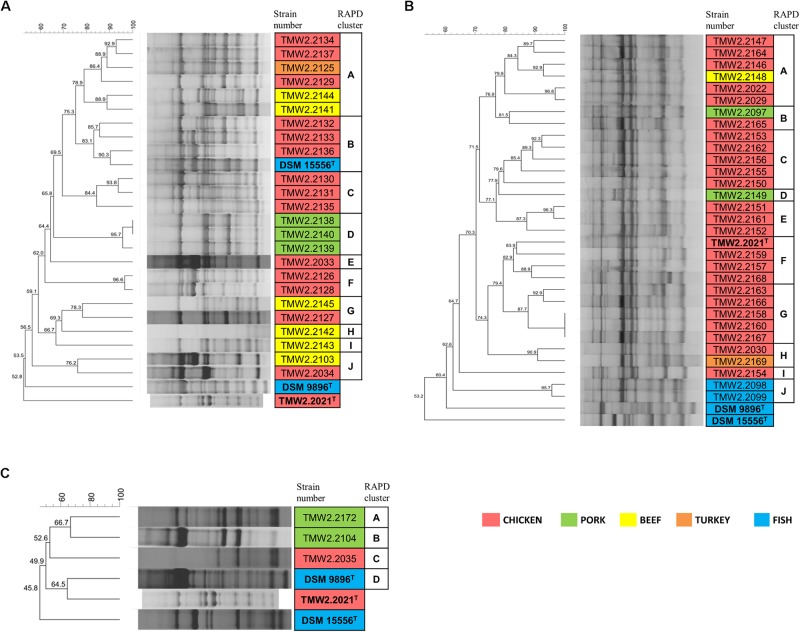
RAPD-clustering of the selected strains. Hierarchical clustering was calculated with the unweighted pair group method with arithmetic mean (UPGMA), Dice similarity coefficient, and 1% tolerance. The similarity values are shown at the nodes of the tree. Different colors specify the isolation sources and bold letters the type strains. Further resolution of the most similar *P. phosphoreum* and *P. carnosum* strains RAPD-clustering is shown in Supplementary Figure S2. **(A)** *P. phosphoreum*, type strain DSM 15556^T^, **(B)** *P. carnosum*, type strain TMW2.2021^T^, **(C)** *P. iliopiscarium*, type strain DSM 9896^T^.

Chromosomal RAPD fingerprints of the strains of the three species were subjected to hierarchical cluster analysis and could be affiliated to several separate groups (Figure 1). In rare cases, RAPD pattern was highly similar and had a 100% dice similarity in one RAPD approach using primer M13V with selected isolates. However, in the other two RAPD-PCR approaches with primer M13V, they exhibited different patterns indicating highly similar, but different strains (Supplementary Figure S2). Furthermore, patterns obtained with additional primer M14V validated their distinctness (Supplementary Figure S3).

Additional analysis of triplicates of all strains confirmed that they cluster together and apart from triplicates of the closest related strains in each case, indicating that respective replicates of one strain were more similar to each other than to other strains. The cluster similarity (dice coefficient) of the triplicates of each strain was at least 3.7% (*P. phosphoreum*), 3.9% (*P. carnosum*), and 22.7% (*P. iliopiscarium*) different from the cluster similarity of triplicates of the respective closest related strain.

Both *P. phosphoreum* and *P. carnosum* strains separated in 10 groups with a threshold of 76 and 79.5% similarity, respectively. Compared to this, the three strains of *P. iliopiscarium* clustered with lower similarity (≤66.7%). Strains from the same meat type did not form coherent cluster, except of *P. phosphoreum* strains from pork and the *P. carnosum* strains from fish. We additionally performed a cluster analysis of all strains of the three species with both primers M13V and M14V (Supplementary Figures S4, S5). All strains from one species cluster together and apart from strains of the other two species thus validating our approach.

### Physiological Differentiation

**FIGURE 2:**
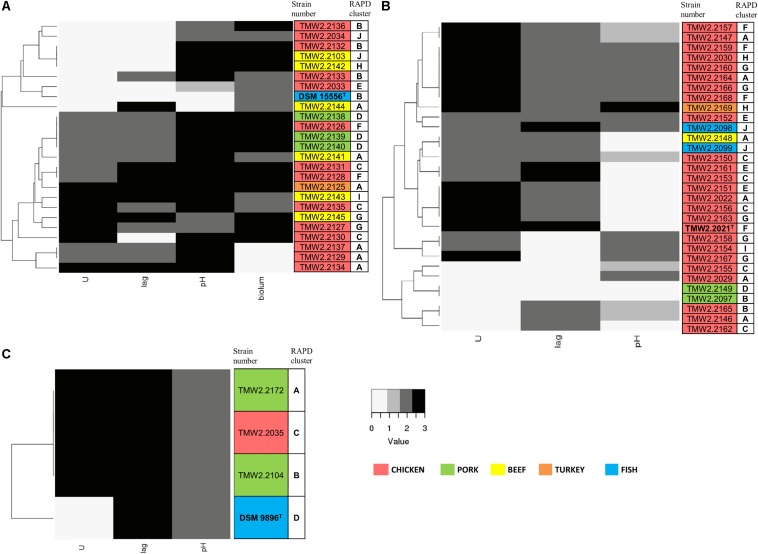
Clustering of the strains by their growth rate (U), lag phase (lag), influence on pH, and bioluminescence (biolum, if existing). Growth rate and lag phase were determined in meat simulation medium at 4°C. Mean values were statistically grouped as low/medium/high and scored from 0 to 3. The highest score was given to the highest growth rates and lowest lag phases, respectively, and a score of 2 was given to medium values. The pH was measured when the cultures reached their highest OD_600_ and scored as follows: 0 – pH ≤5.7, 1 – pH 5.7–5.9, 2 – pH 5.9–6.8, 3 – pH >6.8. Bioluminescence of *P. phosphoreum* was evaluated manually on agar plates. The highest score was given to the strongest bioluminescence. The different isolation sources are displayed in colors and type strains are marked with bolt letters. **(A)** *P. phosphoreum*, type strain DSM 15556^T^, **(B)** *P. carnosum*, type strain TMW2.2021, **(C)** *P. iliopiscarium*, type strain DSM 9896^T^.

We furthermore performed physiotyping experiments to correlate the identified genome-based diversity in relation to phenotypic traits. For that, we monitored the maximum OD_600_, maximum growth rate (U), and lag phase (lag) at 4°C in meat simulation medium at pH 5.8 to mimic cold storage of meats (Table 3). For both – lag phase and maximum growth rate – we could classify the strains in three statistically (*p* < 0.05) different groups within each of the species, and scores were assigned to each of them: short (3), medium (2), and long (0) lag phase and fast (3), medium (2), and slow (0) maximum growth rate. In the case of the pH, since its change is closely related to the production of spoilage substances like biogenic amines, the strains were classified in four groups as they decrease the pH (≤5.7, score 0), leave it unchanged (5.7–5.9, score 1), increase it up to 1 unit (5.9–6.8, score 2), or highly increase it (≥6.8, score 3). The behavior in the medium indicates highly diverse physiotypes that were independent of the isolation source (Figure 2).

**TABLE 3:** Growth parameters of *Photobacterium* spp. in meat-simulation media at 4°C.

**Species**	**Maximum**	**Maximum growth**	**Lag phase**	**pH**
	**OD_600_**	**rate**	**(h)**	
*P. phosphoreum*	3.10–4.99	0.168–0.468	21.17–55.08	5.62–7.47
*P. iliopiscarium*	1.38–2.05	0.033–0.144	32.76–41.83	6.32–6.56
*P. carnosum*	1.36–1.71	0.019–0.061	46.97–101.14	5.43–7.08

*Summary of values obtained for the maximum OD_600_, maximum growth rate (U), lag phase, and pH at maximum OD_600_ during growth of the three species of photobacteria in meat-simulation media at 4°C.*

*Photobacterium phosphoreum* strains reached the highest maximum OD_600_ (up to 4.99), had significantly higher growth rates than *P. carnosum* and *P. iliopiscarium* (*p*-values < 0.05), and tended to increase the pH to a considerable extent (up to pH 7.47). In contrast, *P. carnosum* strains grew up to comparatively low maximum OD_600_ (up to 1.7), had 10 times lower growth rates, and tended to decrease or keep the initial pH value. The only exception was strain TMW2.2169 that alkalized the medium to 7.08. The different influence of the species on the pH was statistically confirmed (*p*-values < 0.05); however, both species included strains that alkalized or acidified the medium at maximum OD_600_. Regarding the lag phase, *P. carnosum* strains adapted to the media approximately half as fast as *P. phosphoreum* strains. Its average lag phase of 47–101 h was significantly longer than the one of both *P. phosphoreum* (21–55 h) and *P. iliopiscarium* (33–42 h, *p*-values < 0.05). The average lag phase of *P. iliopiscarium* was comparable to *P. phosphoreum* (*p*-value 0.767) whereas its maximum growth rate was comparable to *P. carnosum* (*p*-value 0.189). However, the tendency of *P. iliopiscarium* strains to increase the pH only slightly at its maximum OD_600_ (pH 6.32–6.56) was significantly different from the other two species (*p*-values < 0.05).

We observed no general correlation of the growth parameters with the RAPD fingerprint and the isolation source (Figure 2). Nevertheless, *P. phosphoreum* and *P. iliopiscarium* type strains from marine habitats were one of the slowest growing strains of each species, respectively.

Furthermore, *P. iliopiscarium* type strain and additional four *P. phosphoreum* strains from chicken (TMW2.2127, TMW2.2129, TMW2.2130, and TMW2.2134) showed motility after 3 days incubation. The rest of the strains, together with all strains from *P. carnosum*, were non-motile after 3 days. Bioluminescence was a frequent trait of the selected *P. phosphoreum* strains and several meat-borne strains exhibited much higher luminescence than the type strain. Only three *P. phosphoreum* strains from chicken (TMW2.2137, TMW2.2129, and TMW2.2134) did not show bioluminescence at all.

### Resistance to Antibiotics

**FIGURE 3:**
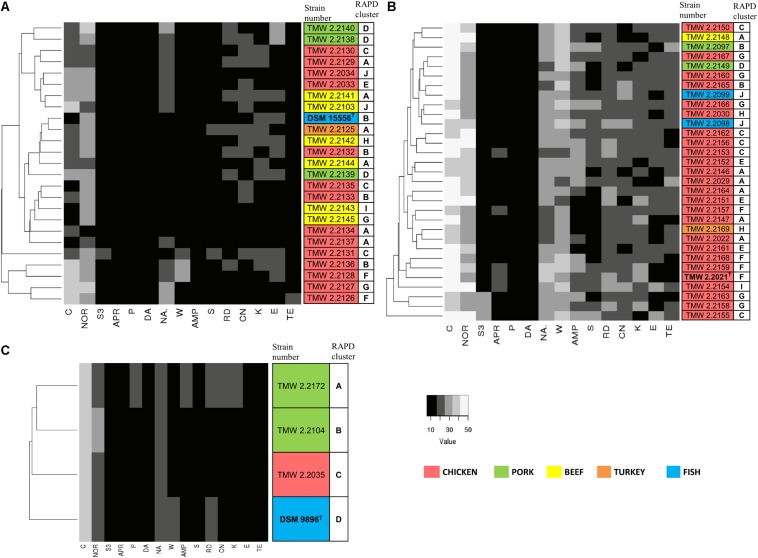
Clustering of the strains by their antibiotic resistance. Resistance was assessed as the diameter of the inhibition zone in mm, with 6 mm being the blank value. C, chloramphenicol (30 μg); NOR, norfloxacin (10 μg); S3, sulfonamides (300 μg); APR, apramycin (25 μg); P, penicillin G (5 μg); DA, clindamycin (2 μg); NA, nalidixic acid (30 μg); W, trimethoprim (5 μg); AMP, ampicillin (10 μg); S, streptomycin (25 μg); RD, rifampicin (5 μg); CN, gentamycin (10 μg); K, kanamycin (30 μg); E, erythromycin (15 μg); TE, tetracycline (30 μg). The different isolation sources are displayed in colors and type strains are marked with bolt letters. **(A)** *P. phosphoreum*, type strain DSM 15556^T^, **(B)** *P. carnosum*, type strain TMW2.2021^T^, **(C)** *P. iliopiscarium*, type strain DSM 9896^T^.

We recorded the tolerance of the strains for 15 antibiotics by measuring their inhibition zones (Table 4 and Figure 3) to evaluate possible correlations between genotypes, isolation sources, and antibiotic resistances. In general, we observed high resistance in almost all strains to clindamycin, apramycin, penicillin G, and sulfonamides but sensitivity to chloramphenicol and norfloxacin. However, a few strains of *P. phosphoreum* exhibited resistance against chloramphenicol and norfloxacin (Figure 3A and Supplementary Table S2). In case of antibiotics with various extent of inhibition, the strains tended to be distributed to either low/high (*P. phosphoreum*) or low/medium/high resistance (*P. carnosum* and *P. iliopiscarium*). *P. carnosum* appeared to be the most sensitive species comprising the highest number of sensitive strains, especially regarding rifampicin, ampicillin, and tetracycline (Supplementary Table S3 and Figure 3B). *P. iliopiscarium* strains appeared to be more similar to the *P. phosphoreum* group than to the *P. carnosum* group regarding resistance to antibiotics (Figure 3C and Supplementary Table S4). Within the species, we did not observe an explicit correlation of antibiotic resistance and isolation source or RAPD clustering. The same applied to the remarkable resistance of some *P. phosphoreum* strains for chloramphenicol and norfloxacin. Furthermore, the type strains revealed no clear differentiation compared to the other strains of the species.

**TABLE 4:** Range diameter of the inhibition zones (mm) as summary of all isolates per species.

**Species**	**DA**	**NOR**	**NA**	**AMP**	**S3**	**W**	**P**	**S**	**APR**	**RD**	**CN**	**K**	**C**	**E**	**TE**
*P. carnosum*	6	24–46	18–40	6–32	6–32	16–38	6–10	6–28	6–18	16–32	12–30	10–30	36–50	6–26	6–26
*P. iliopiscarium*	6	20–24	18–19	6–15	6	6–20	6–15	9–13	6–10	11–18	11–16	10–16	33–35	6–10	6–10
*P. phosphoreum*	6	14–34	8–25	6–12	6–22	6–26	6–12	6–18	6–11	9–22	7–23	6–22	6–38	7–25	6–21

*A diameter of 6 mm was regarded as no inhibition at all. C, chloramphenicol 30 μg; NOR, norfloxacin 10 μg; S3, sulfonamides 300 μg; APR, apramycin 25 μg; P, penicillin G 5 μg; DA, clindamycin 2 μg; NA, nalidixic acid 30 μg; W, trimethoprim 5 μg; AMP, ampicillin 10 μg; S, streptomycin 25 μg; RD, rifampicin 5 μg; CN, gentamycin 10 μg; K, kanamycin 30 μg; E, erythromycin 15 μg; TE, tetracycline 30 μg.*

### Metabolic Properties of Representative Strains

**FIGURE 4:**
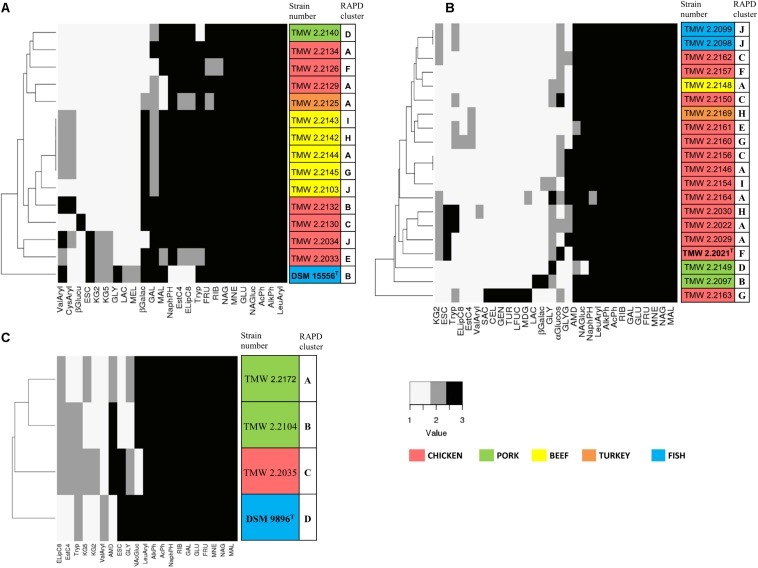
Clustering of selected strains by metabolic properties based on API 50CH and API ZYM. The reactions prove acid production from single carbohydrates (API 50CH) and presence of selected enzymes (API ZYM). Each clustering shows only reactions that were positive at least in one isolate. Reactions were scored as 1 (negative reaction), 2 (weak positive reaction), and 3 (strong positive reaction). The different isolation sources are displayed in colors and type strains are marked with bolt letters. **(A)** *P. phosphoreum*, type strain DSM 15556^T^, **(B)** *P. carnosum*, type strain TMW2.2021^T^, **(C)** *P. iliopiscarium*, type strain DSM 9896^T^. API50ch: GLY, glycerol; RIB, D-ribose; GAL, D-galactose; GLU, D-glucose; FRU, D-fructose; MNE, D-mannose; MDG, methyl-αD-glucopyranoside; NAG, *N*-acetylglucosamine; ESC, esculin; CEL, D-cellobiose; MAL, D-maltose; LAC, D-lactose; MEL, D-melibiose; SAC, D-saccharose; AMD, starch; GLYG, glycogen; GEN, gentiobiose; TUR, D-turanose; LFUC, L-fucose; KG2, potassium 2-ketogluconate; KG5, potassium 5-ketogluconate. APIzym: AlkPh, alkaline phosphatase; EstC4, esterase (C4); ELipC8, esterase lipase (C8); LeuAryl, leucine arylamidase; ValAryl, valine arylamidase; CysAryl, cystine arylamidase; Tryp, trypsin; AcPh, acid phosphatase; NaphPH, naphthol-AS-BI-phosphohydrolase; βGalac, β-galactosidase; βGlucu, β-glucuronidase; αGlucos, α-glucosidase; NAG, *N*-acetyl-β-glucosaminidase.

Biochemical API 50CH and API ZYM tests were conducted with 20 strains of *P. carnosum*, 15 strains of *P. phosphoreum*, and 3 strains of *P. iliopiscarium* in order to study metabolic versatility (Figure 4). All three species produced acid from glucose, mannose, fructose, ribose, and *n*-acetylglucosamine. Additionally, they all responded positively in the tests for alkaline phosphatase, acid phosphatase, and leucine arylamidase. None of the strains produced acid from erythritol, D-arabinose, L-arabinose, D-xylose, L-xylose, D-adonitol, methyl-bD-xylopyranoside, L-sorbose, L-rhamnose, dulcitol, inositol, D-mannitol, D-sorbitol, methyl-α-D-mannopyranoside, amygdalin, arbutin, salicin, D-trehalose, inulin, D-melezitose, D-raffinose, xylitol, D-lyxose, D-tagatose, D-fucose, D-arabitol, and L-arabitol. None of the strains responded positively in the tests for lipase C14, chymotrypsin, α-galactosidase, β-glucosidase, α-mannosidase, and α-fucosidase.

Still, we identified some traits that differed between the species (Supplementary Table S5). Several *P. carnosum* strains produced acid from methyl-α-D-glucopyranoside, cellobiose, saccharose, glycogen, gentiobiose, turanose, and L-fucose in contrast to *P. phosphoreum* and *P. iliopiscarium* strains. *P. carnosum* was additionally the only species with positive or weak positive reactions in the test for α-glucosidase but without acid production from potassium 5-ketogluconate. Strains of *P. phosphoreum* were the only ones being positive for cystine arylamidase and β-glucuronidase and also the only ones that did not produce acid from starch. In contrast, *P. iliopiscarium* strains did not show any unique spectrum of acid production from carbohydrates or enzymatic reactions within the tests. Overall, *P. carnosum* strains covered the broadest carbohydrate utilization spectrum and *P. phosphoreum* strains the most positive enzymatic reactions of all three species.

Within the species, the differences of the marine type strains *P. phosphoreum* DSM15556^T^ and *P. iliopiscarium* DSM9896^T^ to the meat-borne strains were particularly notable. We observed three enzymatic tests that were negative in *P. phosphoreum* DSM15556^T^ but at least weakly positive in all the other *P. phosphoreum* strains (C4 esterase, C8 esterase–lipase, naphthol-AS-BI-phosphohydrolase; Supplementary Table S5). On the other hand, three carbohydrates were exclusively used by the type strain for acid production (glycerol, D-lactose, and D-melibiose). We saw also three reactions that were different for *P. iliopiscarium* DSM9896^T^ compared to meat-borne *P. iliopiscarium* strains (C8 esterase–lipase, valine arylamidase, and starch metabolism).

Furthermore, we identified a correlation of isolation source and metabolic properties that was depicted by the clustering of almost all *P. phosphoreum* strains from beef (Figure 4A). However, we could not identify clear differences of *P. carnosum* strains from meat and *P. carnosum* strains from fish (Supplementary Table S5). The test results of the *P. carnosum* type strain TMW2.2021^T^ were also not clearly different when compared to the other meat-borne strains. Nevertheless, both *P. carnosum* strains from salmon cluster together and both strains from pork cluster apart from the rest (Figure 4B). In each species we observed some reactions that were solely positive in single strains. *P. carnosum* TMW2.2163 was the only strain producing acid from saccharose, cellobiose, gentiobiose, turanose, and L-fucose (Figure 4B). *P. phosphoreum* TMW2.2130 was conspicuous by β-glucuronidase activity and *P. iliopiscarium* TMW2.2035 by acid production from potassium 2-ketogluconate (Figures 4A,C).

## Discussion

This is the first study that investigated biodiversity of meat-borne isolates of *Photobacterium* spp., isolated a wide variety of strains and explored strain- as well as species-specific traits. The data obtained from our study give further evidence that photobacteria, specifically *P. phosphoreum*, *P. carnosum*, and *P. iliopiscarium*, are widespread contaminants of different meats, as previously stated in Hilgarth et al. (2018a).

### Distribution of *Photobacterium* spp. Contaminants

Recently, reports on the presence of photobacteria have emerged, mostly in culture-independent studies without actual isolation. All these reports were widespread over different countries, i.e., Germany (Hilgarth et al., 2018a), Belgium (Stoops et al., 2015), Italy (Pennacchia et al., 2011), Denmark (Nieminen et al., 2016), France (Bouju-Albert et al., 2018), and China (Li et al., 2019), demonstrating the global relevance of photobacteria to meat spoilage. Together with this, the data of our study confirm that contamination of meat with *Photobacterium* spp. is not sporadic, but rather a general issue associated with the meat industry. They also suggest that the contamination source might be similar in all types of meat, and therefore should be located in a common part of the slaughtering, processing, or packaging of the meat. This would also allow speculation on the presence of photobacteria associated with livestock, prior to the slaughtering process. However, given the psychrophilic nature of these organisms, and the inability of *P. carnosum* to grow at temperatures >20°C, or *P. phosphoreum* and *P. iliopiscarium* >25°C (Hilgarth et al., 2018b), it appears unlikely that these bacteria are autochthonous members of the animal gut-microbiome. Furthermore, we did not recover any photobacteria from other animal-derived products besides meat, nor from MAP packed-, protein- rich-, or sea-related vegetables. This suggests that, in relationship to food contamination and spoilage, photobacteria seem to only be able to reach detectable numbers on meat (and fish). We also did not detect photobacteria on two types of seafood (scallops and shrimps). However, these products had been deep-frozen before sampling and it has been reported that deep-freezing reduces photobacteria below detection limits for culture-dependent methods (Emborg et al., 2002; Dalgaard et al., 2006).

### Occurrence and Diversity of *Photobacterium* spp. on Packaged Meats

Calculated rarefaction and diversity indices revealed that the large quantity of isolates analyzed in this study reflects expected abundances. It therefore allows representative assessment of diversity within and between the species *P. carnosum* (31 strains from 163 isolates) and *P. phosphoreum* (24 strains from 113 isolates). The high evenness of *P. phosphoreum* and *P. carnosum* strains demonstrate the absence of dominant genotypes and suggest a rather general adaptation of the strains. However, even strains from the same meat sample showed clear genotypic and phenotypic variability, which suggests an initial contamination that is already considerably diverse. Furthermore, ecological entropy of both species was not significantly different meaning the same degree of overall biodiversity also on species level. Regarding *P. iliopiscarium*, the low number of recovered isolates (three isolates with three genotypes) suggests that there may be more diversity within the meat-borne strains than the ones recovered in this study.

We did not isolate any photobacteria from either minced beef- or mixed minced meat in this study. However, culture-independent reports of *Photobacterium* spp. (Pennacchia et al., 2011; Stoops et al., 2015) indicate that the genus can be present on minced meat, even if they do not grow to detectable numbers. It may be speculated that other meat spoilers dominate on minced meat and simply overgrow photobacteria due to shorter doubling time. Recently, presence of *Pseudomonas* spp. has been reported on MAP minced meat (Hilgarth et al., 2019) that might act as possible fast growing competitor of *Photobacterium* spp.

We also observed that not all samples of meat cuts are contaminated with photobacteria, even if they come from the same producer. This could indicate a low level of initial contamination and distribution by chance (Höll et al., 2019). A low initial contamination may also explain the different distribution of the three *Photobacterium* species on different meat types (Hilgarth et al., 2018a).

The growth of photobacteria appears also be independent of the packaging method since photobacteria occur independently of the employment of modified atmosphere, vacuum, or air packaging (Hilgarth et al., 2018a). This is supported by Höll et al. (2019) who predicted that there is little to no effect of the choice of atmosphere on the growth of photobacteria, based on similar gene expression under different MAP conditions. This suggests that the current modified atmosphere composition and vacuum packages, commonly used to extend the shelf-life and optimum qualities of meat and fish (McKee, 2007; McMillin, 2008; Bingol and Ergun, 2011; Lorenzo and Gomez, 2012; Rossaint et al., 2014), are insufficient to reduce spoilage-associated photobacteria on meat. Furthermore, the detection of photobacteria on marinated meats demonstrated that marinating – a process to introduce antimicrobials (Björkroth, 2005; Kargiotou et al., 2011) – will also not prevent photobacterial spoilage.

### Adaptation to Food as an Ecological Niche

Results from the carbon metabolism and enzymatic activities, together with distribution of growth rates and lag phase, suggest that *P. carnosum* strains are more homogeneous with lower variability than *P. phosphoreum* and *P. iliopiscarium* strains. While it was possible to clearly differentiate the marine type strain of the two latter from the meat-borne strains, *P. carnosum* seems to share common traits for all the strains, independently of the source of isolation. Additionally, our results for the growth and metabolic traits indicate adaptation of *P. carnosum* to meat or other nutrient rich environments, as stated before by Hilgarth et al. (2018b). *P. carnosum* also lacks bioluminescence and motility, two common traits of symbiotic or free-living marine photobacteria. This supports missing adaptation of the species to sea-related environments. Still, for the first time, *P. carnosum*, a species described as terrestrial and unrelated to sea environments, was detected on MAP salmon. However, our data on missing subpopulations referring to respective environments support the hypothesis that the isolates from (freshwater) farmed salmon do not originate from a marine environment, but rather from a contamination later in the processing and packaging. The fact that *P. phosphoreum* isolates originating from the same MAP farmed salmon showed no distinct genotypes, i.e., were also found on meats, further supports that hypothesis. In contrast, *P. phosphoreum* and *P. iliopiscarium* appear to have different marine as well as meat-borne subpopulations with specific adaptations to the respective environment as demonstrated by the differences of the meat borne strains to their marine type strains.

Reactions for lipase C14, esterase C4, and esterase–lipase C8 were negative or at most weakly positive for almost all strains of the three species. Additionally, all of the *P. phosphoreum* meat-borne strains and some from *P. iliopiscarium* and *P. carnosum* were negative for glycerol. However, Höll et al. (2019) confirmed the expression of lipase and genes encoding for enzymes involved in lipid and glycerol utilization in photobacteria. This suggests that the lipase was not expressed in API medium or that this type of lipase do not lead to a positive reaction within the API ZYM test and that utilization of glycerol does not result in acidification of the medium. However, almost all strains of the three species showed positive reactions for the main monomeric carbohydrates found in meat, i.e., glucose, fructose, mannose, ribose (Aliani and Farmer, 2005a, b; Koutsidis et al., 2008a, b; Meinert et al., 2009a, b). Furthermore, the species *P. carnosum* shows a wider metabolic capability in terms of carbohydrate utilization than the other two species. Many of the carbohydrates used exclusively by *P. carnosum* are plant (e.g., starch, cellobiose, gentiobiose, turanose) or meat related (e.g., glycogen). Regarding growth on meat-simulation media, we observed that the species has the lowest maximum growth rates and longer adaptation times in the meat-simulation media used in this study. However, it is found in some meat types in larger amounts and cell counts than any of the other two species. This suggests that *P. carnosum* is adapted to more complex media and has specific growth requirements that the other two species do not have.

### Safety Concerning Aspects of *Photobacterium* Species

The observed variable alkalization or acidification of the growth medium with up to two pH values difference demonstrates the great variety of strain physiotypes. This might also be of relevance for the respective potential as meat spoiler since alkalization indicates production of biogenic amines and ammonia from amino acid metabolism. The ability of *P. phosphoreum* to produce histamine and other biogenic amines in fish has been previously reported (Jorgensen et al., 2000; Stoops et al., 2015; Nieminen et al., 2016). The increase of pH in the media up to 7.5 might be an indicator for the potential of some of our isolates, i.e., certain strains of *P. phosphoreum* to produce higher amounts of biogenic amines, which is also predicted in the transcriptomic analysis of Höll et al. (2019).

Another important safety aspect deals with bacterial resistance to antibiotics. Administration of antibiotics to poultry, swine, and calves in the agricultural industry is known as disease treatment and control (Nisha, 2008; Muaz et al., 2018) and therefore possibly linked to resistance of meat spoiling bacteria. However, we did not observe a clear pattern that would allow to link the source of isolation to the antibiotic resistances determined in this study. Our results suggest that the species have intrinsic resistance to clindamycin, apramycin, penicillin G, and sulfonamides. However, resistance to the other antibiotics occurs differentially on strain level. The fact that closely related strains with similar chromosomal fingerprints did not exhibit similar antibiotic resistances suggests that these resistances may be located on mobile genetic elements and therefore possibly be transferable. This transferability might also occur for chloramphenicol and norfloxacin resistance in *P. phosphoreum*, as only few of its strains show complete resistance to them in contrast to the common tendency of the three species. The suggested transferability of the resistance to chloramphenicol, being one of the drugs of last resort [DoLR (World-Health-Organization, 2001)], harbors potential health concerns.

## Conclusion

This study demonstrates that, even though the initial contamination is likely to be low, photobacteria strains from meat display a great diversity with specific genotypic, phenotypic, and physiotypic traits. Due to previous association with solely marine environments and lack of optimized detection methods, biodiversity of meat-borne *P. phosphoreum*, *P. iliopiscarium*, and *P. carnosum* was hitherto unexplored. On the basis of our results, we can assume that their entry route as meat contaminants might occur during slaughtering, derived from the exterior of the animal or environment, but not from the gut – following colonization of general processing and packaging facilities. Divergence of the meat-borne and the marine type strains of *P. phosphoreum* and *P. iliopiscarium* on the one hand and homogeneity of *P. carnosum* strains on the other hand suggests different environmental adaptation and possibly also separate origin of contamination. Additionally, diversity of metabolic capabilities and antibiotic resistances appear to be widespread and mostly not linked to a specific isolation source. This reveals the presence of a highly variable and rich community of photobacteria on each meat that combines multiple physio- and genotypes with potential relevance to food safety worldwide.

## Data Availability Statement

All datasets generated for this study are included in the manuscript/Supplementary Files.

## Author Contributions

SF-P and PH performed the laboratory work and data evaluation, wrote the first draft of the manuscript, and designed the study. MH performed the diversity index analysis, helped to draft the study, and supervised the work of SF-P and PH. RV initiated the project and supervised the work of SF-P and PH. All authors read and approved the final manuscript.

## Conflict of Interest

The authors declare that the research was conducted in the absence of any commercial or financial relationships that could be construed as a potential conflict of interest.

## Footnotes

1 www2.heatmapper.ca/expression/
