# Supplementary material for: Biodiversity of Photobacterium spp. Isolated From Meats
Source: Front Microbiol. 2019 Oct 18;10:2399. doi: 10.3389/fmicb.2019.02399 (PMC6842964; doi:10.3389/fmicb.2019.02399)
Supplement: Supplementary file 1 [file Data_Sheet_1.PDF]

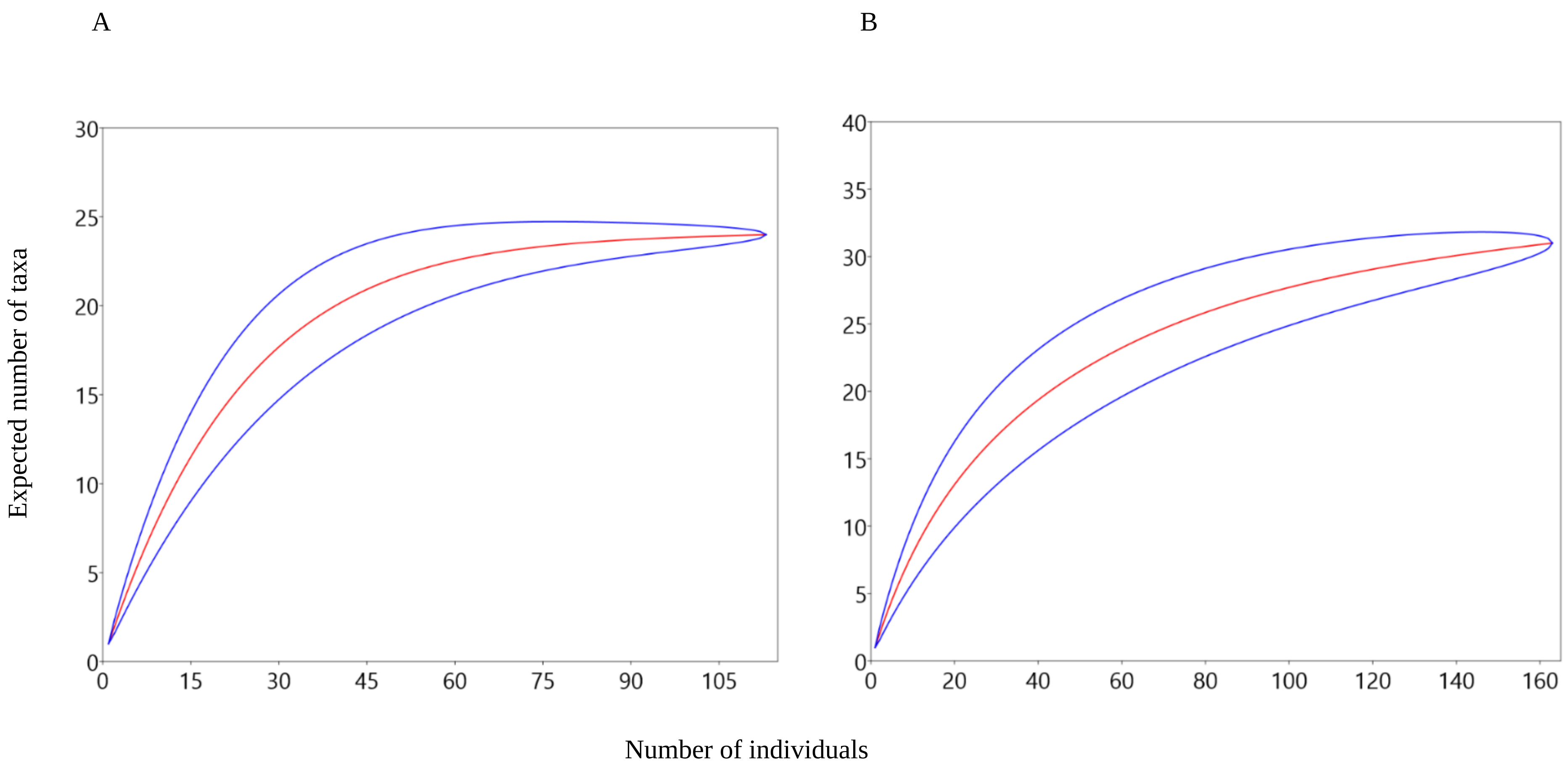

Figure S1 Rarefaction analysis visualization of A. *P. phosphoreum* and B. *P. carnosum*.
Blue line shows 95% confidence interval.

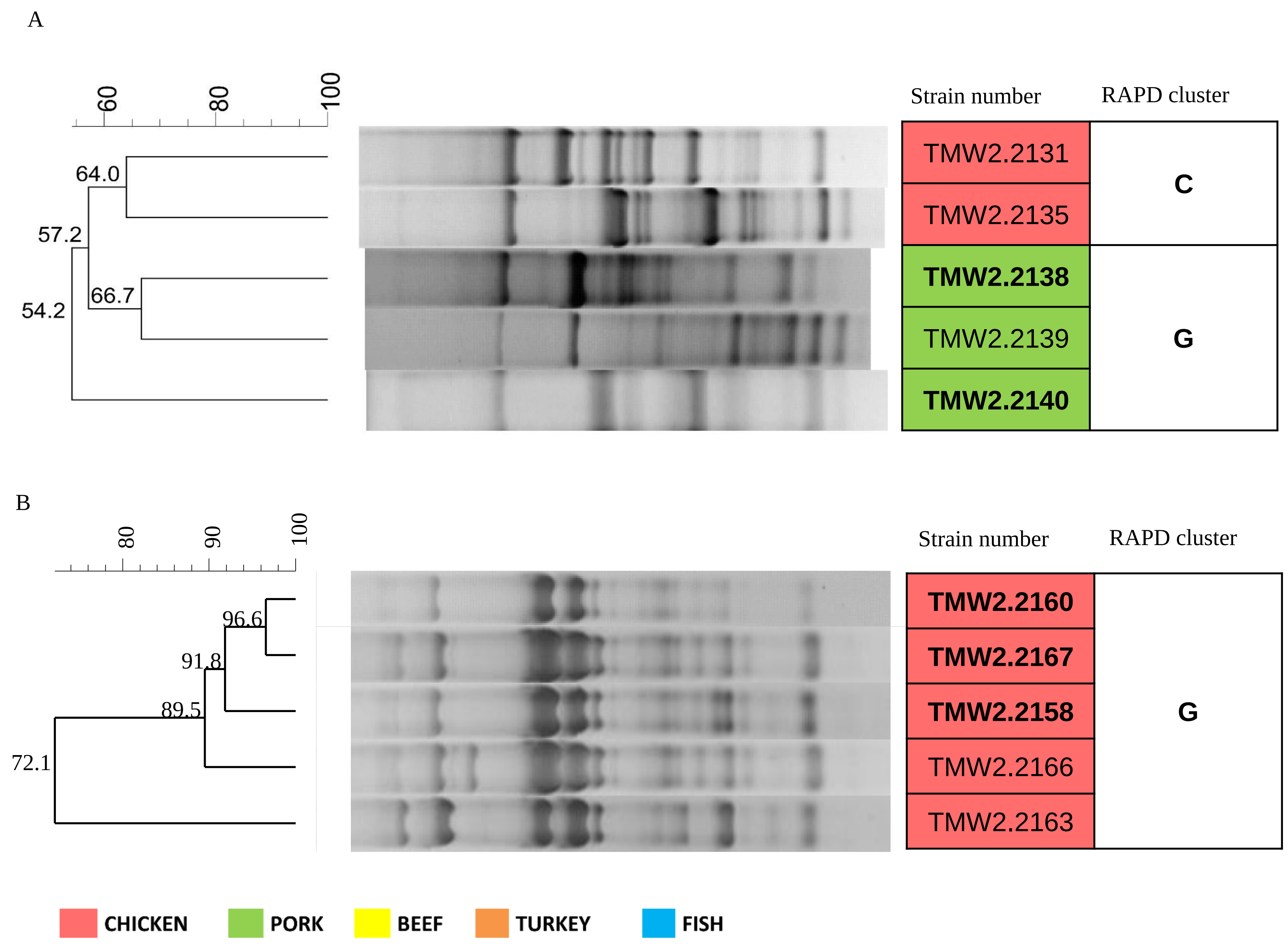

Figure S2 RAPD-clustering of the most similar strains of *P. phosphoreum* and *P. carnosum* used for the preliminary strain selection .
Hierarchical clustering was calculated with the unweighted pair group method with arithmetic mean (UPGMA), Dice similarity coefficient and 1 % tolerance. The RAPD-clustering of all selected isolates in the manuscript (see Fig. 1) shows high similarity of strains TMW2.2138, TMW2.2140 from *P. phosphoreum* and strains TMW2.2160, TMW2.2167, TMW2.2158 from *P. carnosum*, respectively. However, initial comparison of all recovered isolates showed clear differences of the mentioned strains from **A** *P. phosphoreum* and **B** *P. carnosum*. Therefore, isolates were kept for the further study.

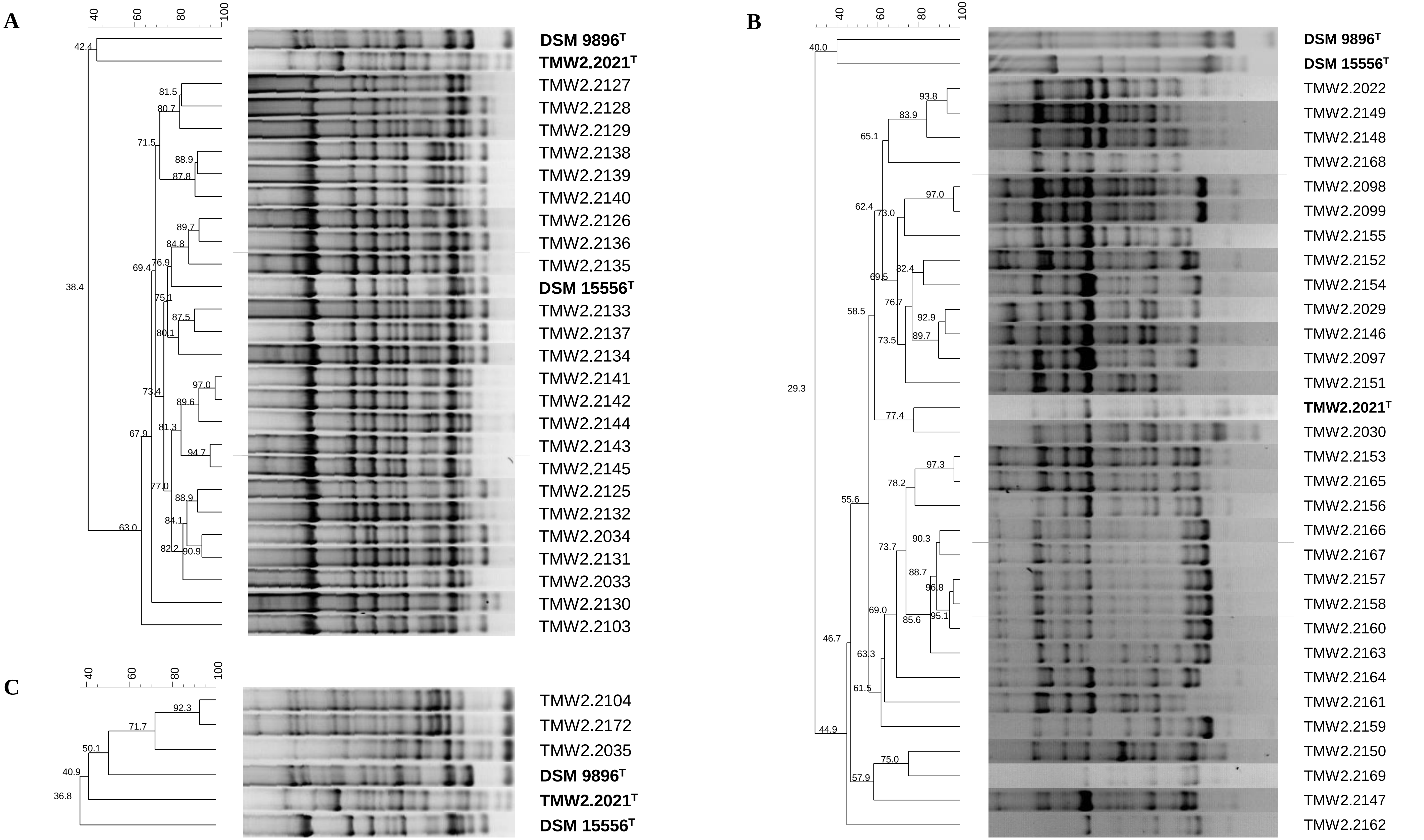

Figure S3 RAPD-clustering of the selected strains with additional primer M14V.
Strain differentiation based on primer M13V was confirmed with additional primer M14V. Hierarchical clustering was calculated with the unweighted pair group method with arithmetic mean (UPGMA), Dice similarity coefficient and 1 % tolerance. The similarity values are shown at the nodes of the tree.
A *P. phosphoreum*, type strain DSM 15556^T, **B** *P. carnosum*, type strain TMW2.2021^T, **C** *P. iliopiscarium*, type strain DSM 9896^T.

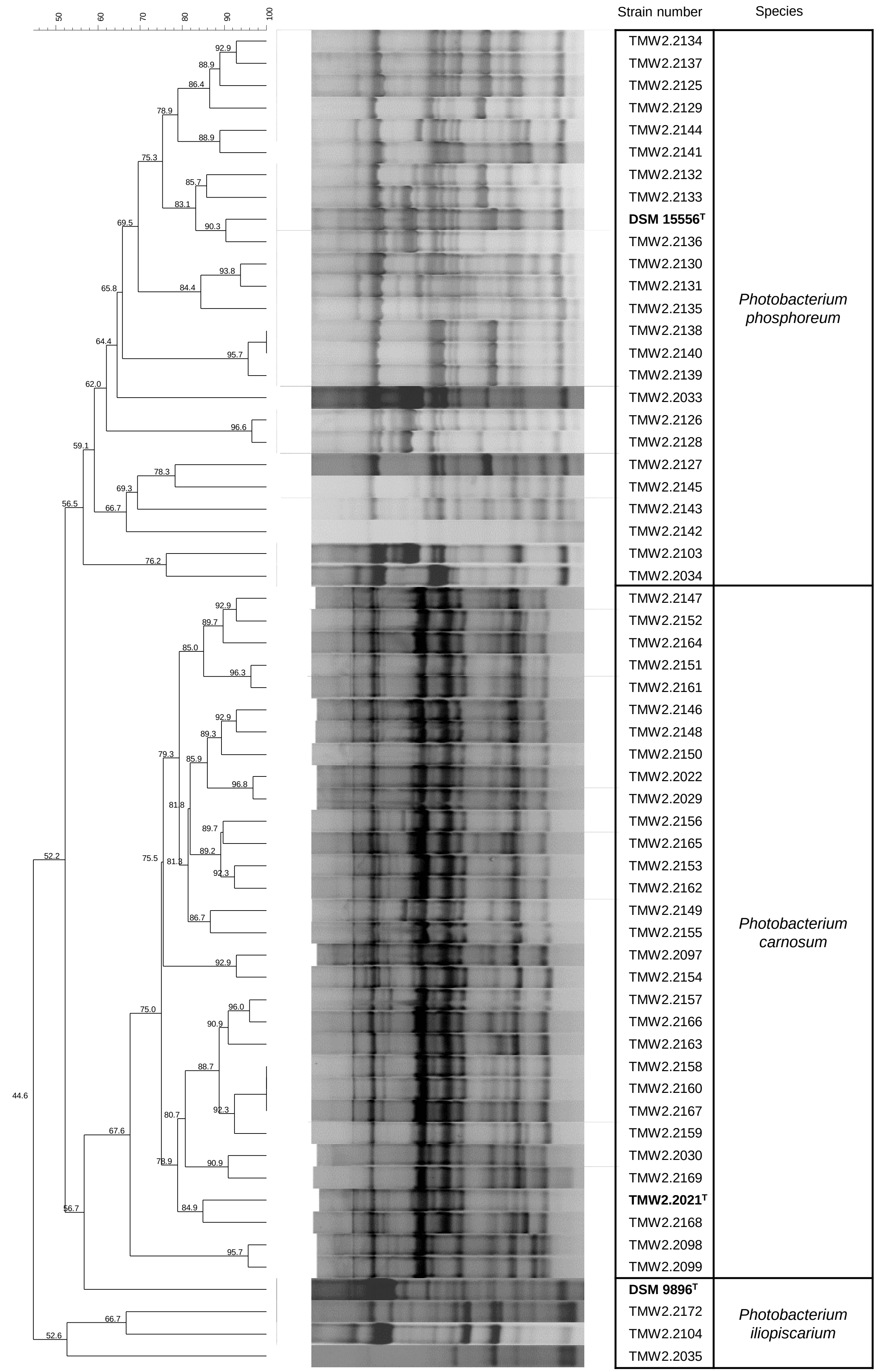

Figure S4 RAPD-clustering of all the selected strains of the three species of photobacteria together.
Hierarchical clustering was calculated with the unweighted pair group method with arithmetic mean (UPGMA), Dice similarity coefficient and 1% tolerance. Similarity values are shown at the nodes of the tree. All strains of one species cluster together, and apart from the strains belonging to another species.

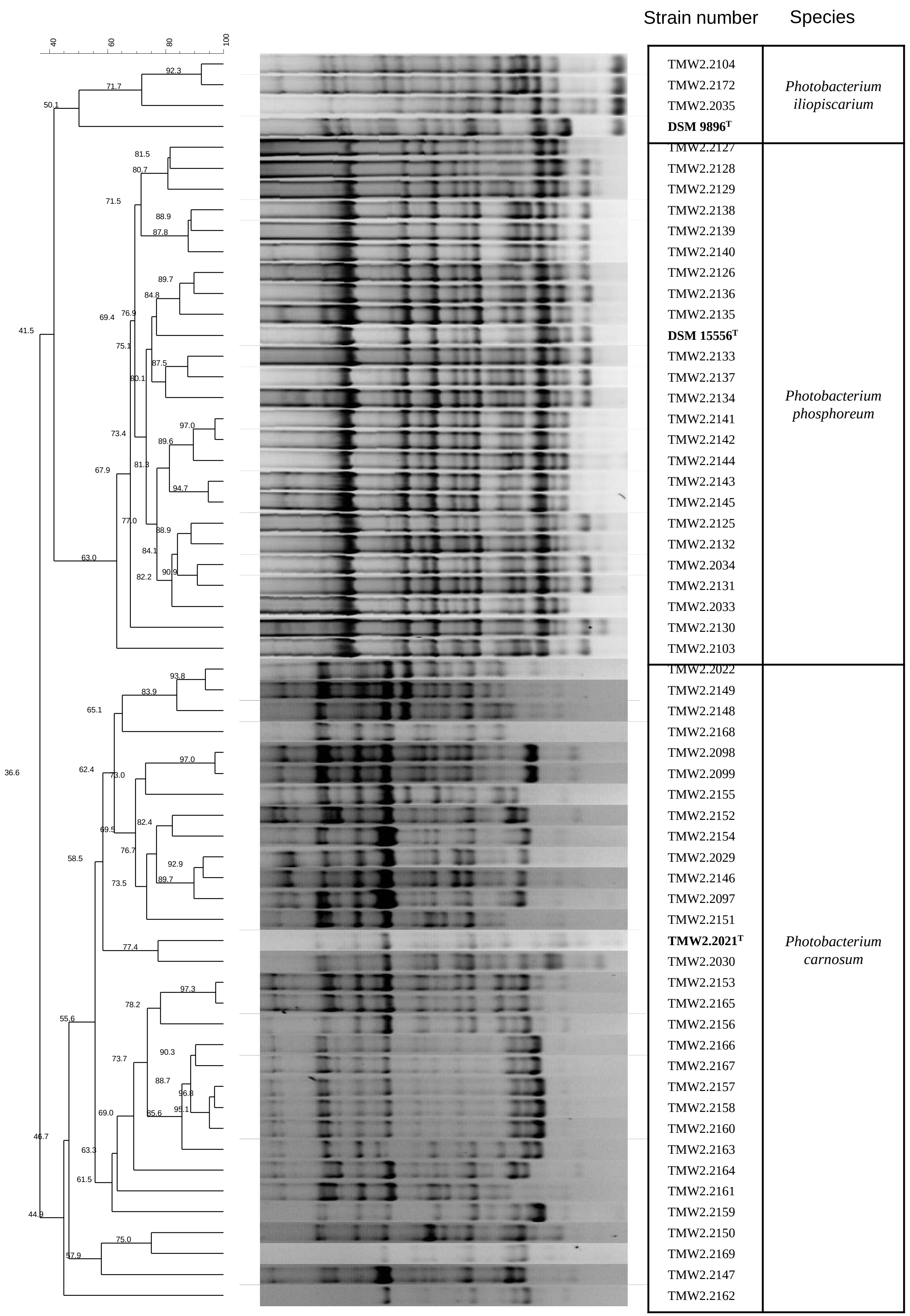

Figure S5 RAPD-clustering of the selected strains of all three species with additional primer M14V.
Species differentiation based on primer M13V was confirmed with additional primer M14V. Hierarchical clustering was calculated with the unweighted pair group method with arithmetic mean (UPGMA), Dice similarity coefficient and 1 % tolerance. The similarity values are shown at the nodes of the tree. Selected strains of all three species were included.
P. phosphoreum, type strain DSM 15556^{T}, *P. carnosum*, type strain TMW2.2021^{T}, *P. iliopiscarium*, type strain DSM 9896^{T}.

Table S1 Strains origin

Origin by type of packaging, type of meat, and sample, of the strains included in the study. The number of isolates screened refers to the number of isolates from that species that were recovered from the sample, and compared by RAPD PCR approach. The number of strains refers to the amount of strains obtained from the recovered isolates.

Package	Meat type	Contaminated/ Sampled	Sample	*P. carnosum* isolates screened	*P. carnosum* strains	TMW	*P. phosphoreum* isolates screened	*P. phosphoreum* strains	TMW	*P. iliopiscarium* isolates screened	*P. iliopiscarium* strains	TMW
MAP	Chicken*	5/15	1	-	-	-	4	2	TMW2.2033 TMW2.2034	1	1	TMW2.2035
			2	21	2	TMW2.2021** TMW2.2022** TMW2.2030** TMW2.2146 TMW2.2147	-	-	-	-	-	-
			3			TMW2.2029**	-	-	-	-	-	-
MAP	Beef*	2/2	1	-	-	-	2	1	TMW2.2103	-	-	-
MAP	Pork*	2/9	1	7	2	TMW2.2097 TMW2.2149	-	-	-	1	1	TMW2.2104
			2	-	-	-	-	-	-	1	1	TMW2.2172
MAP	Marinated chicken	2/3	1	99	15	TMW2.2151 TMW2.2152 TMW2.2153 TMW2.2155 TMW2.2156 TMW2.2157 TMW2.2158 TMW2.2159 TMW2.2160 TMW2.2161 TMW2.2162 TMW2.2163 TMW2.2164 TMW2.2165 TMW2.2166	26	12	TMW2.2134 TMW2.2137 TMW2.2129 TMW2.2132 TMW2.2133 TMW2.2136 TMW2.2130 TMW2.2131 TMW2.2135 TMW2.2127 TMW2.2126 TMW2.2128	-	-	-
			2	27	3	TMW2.2167 TMW2.2168 TMW2.2154	-	-	-	-	-	-
MAP	Marinated beef	1/3	1	-	-	-	35	5	TMW2.2144 TMW2.2141 TMW2.2145 TMW2.2142 TMW2.2143	-	-	-
MAP	Salmon	6/6	1	3	1	TMW2.2099	27	-	-	-	-	-
			2	1	1	TMW2.2098	10	-	-	-	-	-
Air	Chicken	1/4	1	3	1	TMW2.2150	-	-	-	-	-	-
Air	Beef	1/3	1	1	1	TMW2.2148	-	-	-	-	-	-
Air	Pork	1/3	1	-	-	-	7	3	TMW2.2138 TMW2.2139 TMW2.2140	-	-	-
Air	Marinated turkey	1/3	1	1	1	TMW2.2169	2	1	TMW2.2125	-	-	-

*Samples marked belong to the previous study by Hilgarth *et al.* (Hilgarth *et al.*, 2018a).

**Marked strains were obtained fromt he previous work by Hilgarth *et al.* (Hilgarth *et al.*, 2018b), from the *P. carnosum* sp. nov. species description.

Table S2. Antibiotic inhibition zone of *P. phosphoreum*.

Diameter values in mm of the inhibition zone observed for every antibiotic and each of the selected isolates of *P. phosphoreum*. The diameter of the antibiotic discs was measured as 6 mm, and therefore values of 6 in the table represent no inhibition zone observed.

Strain	Clindamycin DA 2 µg	Norfloxacin NOR 10 µg	Nalidixic acid NA 30 µg	Ampicillin AMP 10 µg	Sulphonamides S3 300 µg	Trimetoprim W 5 µg	Penicillin G P 5 µg	Streptomycin S 25 µg	Apramycin APR 15 µg	Rifampicin RD 5 µg	Gentamycin CN 10 µg	Kanamycin K 30 µg	Chloramphenicol C 30 µg	Erythromycin E 15 µg	Tetracyclin TE 30 µg
**TMW 2.2103**	6	22	19	6	6	6	6	10	6	13	15	14	35	13	6
**TMW 2.2033**	6	25	20	6	6	6	6	11	8	12	15	14	31	10	7
**TMW 2.2034**	6	25	20	6	6	6	6	10	6	11	14	13	27	8	6
**TMW 2.2126**	6	32	25	8	6	6	6	7	6	14	7	6	36	11	21
**TMW 2.2127**	6	34	25	6	6	6	6	10	6	13	8	6	38	11	6
**TMW 2.2128**	6	25	22	6	6	25	6	8	6	12	10	7	36	17	6
**TMW 2.2138**	6	25	20	11	6	6	6	13	11	18	16	14	20	24	6
**TMW 2.2139**	6	32	9	12	6	6	12	12	9	15	14	16	25	21	6
**TMW 2.2140**	6	33	25	11	6	6	10	11	10	17	10	12	20	25	6
**TMW 2.2125**	6	30	10	6	6	6	6	16	11	15	17	20	16	14	6
**DSM 15556^T**	6	29	9	6	6	6	6	12	10	13	13	20	13	15	6
**TMW 2.2141**	6	26	20	6	6	6	6	12	6	16	18	16	30	15	6
**TMW 2.2142**	6	25	10	6	6	6	6	12	10	12	23	20	15	17	6
**TMW 2.2143**	6	24	8	6	6	6	6	13	6	11	11	9	12	8	6
**TMW 2.2144**	6	21	9	6	6	6	6	10	10	14	14	22	18	11	6
**TMW 2.2145**	6	28	8	6	6	6	6	9	11	12	10	6	6	9	6
**TMW 2.2129**	6	21	20	6	6	6	6	14	8	15	18	15	23	12	6
**TMW 2.2130**	6	22	20	10	6	6	6	12	6	13	15	15	17	8	6
**TMW 2.2131**	6	31	8	6	22	23	6	18	11	10	19	20	16	15	6
**TMW 2.2132**	6	25	9	6	6	6	6	9	10	16	16	14	17	17	6
**TMW 2.2133**	6	26	8	6	6	6	6	8	6	13	15	11	15	10	6
**TMW 2.2134**	6	14	11	8	6	6	6	12	9	9	12	6	16	8	6
**TMW 2.2135**	6	26	8	9	6	6	6	6	10	11	17	13	13	7	6
**TMW 2.2136**	6	28	18	6	9	26	6	14	10	22	14	18	37	22	6
**TMW 2.2137**	6	21	18	6	6	6	6	6	9	12	12	11	6	8	6

Table S3. Antibiotic inhibition zone of *P. carnosum*.

Diameter values in mm of the inhibition zone observed for every antibiotic and each of the selected isolates of *P. carnosum*. The diameter of the antibiotic discs was measured as 6 mm, and therefore values of 6 in the table represent no inhibition zone observed.

Strain	Clindamycin DA 2 µg	Norfloxacin NOR 10 µg	Nalidixic acid NA 30 µg	Ampicillin AMP 10 µg	Sulphonamides S3 300 µg	Trimetoprim W 5 µg	Penicillin G P 5 µg	Streptomycin S 25 µg	Apramycin APR 15 µg	Rifampicin RD 5 µg	Gentamycin CN 10 µg	Kanamycin K 30 µg	Chloramphenicol C 30 µg	Erythromycin E 15 µg	Tetracyclin TE 30 µg
**TMW 2.2021[T]**	6	26	40	20	6	28	6	20	16	26	20	30	46	12	12
**TMW 2.2022**	6	32	18	18	6	28	6	14	6	16	20	18	38	14	18
**TMW 2.2029**	6	36	28	32	6	16	10	20	6	22	22	14	46	8	26
**TMW 2.2030**	6	32	24	20	6	32	6	22	12	20	20	20	44	10	18
**TMW 2.2098**	6	36	26	24	6	36	6	22	6	24	24	16	40	16	22
**TMW 2.2099**	6	32	28	18	6	30	6	16	10	22	26	20	42	14	20
**TMW 2.2097**	6	38	28	24	6	36	6	28	6	20	20	18	50	12	24
**TMW 2.2146**	6	36	26	28	6	26	10	14	6	20	12	18	40	6	20
**TMW 2.2147**	6	28	22	10	6	24	6	14	10	20	16	28	42	6	14
**TMW 2.2148**	6	44	24	20	6	38	6	14	6	20	12	12	44	20	20
**TMW 2.2149**	6	32	22	26	6	36	6	12	6	18	14	16	44	18	20
**TMW 2.2150**	6	40	30	18	6	34	6	18	6	16	14	18	48	16	22
**TMW 2.2151**	6	44	26	6	6	20	6	12	6	24	12	24	44	18	16
**TMW 2.2152**	6	36	26	30	6	24	6	16	6	22	16	10	40	20	16
**TMW 2.2153**	6	40	22	24	6	22	6	22	16	26	18	22	44	16	18
**TMW 2.2154**	6	44	28	22	6	34	6	18	16	30	30	20	50	26	22
**TMW 2.2155**	6	46	30	24	32	38	6	24	6	18	18	14	48	24	20
**TMW 2.2156**	6	42	24	26	6	30	6	20	6	24	18	16	42	16	6
**TMW 2.2157**	6	24	20	6	6	28	6	14	16	22	18	20	36	16	18
**TMW 2.2158**	6	26	24	22	30	24	6	16	14	24	18	24	40	14	20
**TMW 2.2159**	6	40	38	26	6	38	6	22	18	18	22	24	50	14	6
**TMW 2.2160**	6	24	26	20	6	32	6	12	10	22	20	16	42	16	20
**TMW 2.2161**	6	38	24	6	6	32	6	12	6	20	12	16	42	22	18
**TMW 2.2162**	6	44	24	22	6	32	6	20	12	22	18	20	40	16	12
**TMW 2.2163**	6	26	26	24	26	32	6	14	6	16	18	16	38	14	6
**TMW 2.2164**	6	36	26	16	6	24	6	6	6	32	16	16	42	22	18
**TMW 2.2165**	6	32	22	20	6	36	6	12	12	18	26	20	42	16	18
**TMW 2.2166**	6	32	26	24	6	34	6	24	12	20	16	18	38	18	18
**TMW 2.2167**	6	30	26	22	6	34	6	16	6	22	18	14	44	20	20
**TMW 2.2168**	6	28	28	28	6	36	6	14	12	20	18	16	48	16	6
**TMW 2.2169**	6	32	22	6	6	26	6	14	6	18	18	16	40	14	18

Table S4. Antibiotic inhibition zone of *P. iliopiscarium*.

Diameter values in mm of the inhibition zone observed for every antibiotic and each of the selected isolates of *P. iliopiscarium*. The diameter of the antibiotic discs was measured as 6 mm, and therefore values of 6 in the table represent no inhibition zone observed.

Strain	Clindamycin DA 2 µg	Norfloxacin NOR 10 µg	Nalidixic acid NA 30 µg	Ampicillin AMP 10 µg	Sulphonamides S3 300 µg	Trimetoprim W 5 µg	Penicillin G P 5 µg	Streptomycin S 25 µg	Apramycin APR 15 µg	Rifampicin RD 5 µg	Gentamycin CN 10 µg	Kanamycin K 30 µg	Chloramphenicol C 30 µg	Erythromycin E 15 µg	Tetracyclin TE 30 µg
**DSM 9896^T**	6	20	18	9	6	20	6	10	6	15	12	13	34	6	10
**TMW 2.2035**	6	20	18	6	6	6	6	9	6	11	12	10	33	10	6
**TMW 2.2104**	6	24	19	14	6	6	6	9	10	14	11	11	35	6	8
**TMW 2.2172**	6	20	19	15	6	6	15	13	9	18	16	16	33	10	6

Table S5. Comparison of positive metabolic reactions in API50ch and APIzym between type strain and the rest of isolates of the species.

Summary of the positive reactions found in the selected *Photobacterium* strains. For each species, the table shows the results recorded for the type strain, and the results observed in at least one of the other strains of the species. Marked in light red are the differences observed between each of the type strains and the rest of the strains. In the case of *P. phosphoreum* and *P. iliopiscarium*, it additionally represents the differences between the sea-related type strain and the meat-related strains. Positive reactions are marked with a „+“ sign, negative reactions are marked with a „-“ sign, while weakly positive reactions are marked with a „w“.

Reaction	*P. phosphoreum*		*P. carnosum*		*P. iliopiscarium*	
	DSM 15556^T	Species	TMW 2.2021^T	Species	DSM 9896^T	Species
**Alkaline phosphatase**	+	+	+	+	+	+
**Esterase (C 4)**	-	+/w	-	w/-	-	w/-
**Esterase Lipase (C 8)**	-	+/w	-	w/-	-	w
**Leucine arylamidase**	+	+	+	+	+	+
**Valine arylamidase**	+	+/w/-	-	w/-	w	-
**Cystine arylamidase**	-	+/w/-	-	-	-	-
**Trypsin**	+	+/w/-	-	+/w/-	w	w/-
**Acid phosphatase**	+	+	+	+	+	+
**Naphthol-AS-BI-phosphohydrolase**	-	+	+	+/w	+	+
**β-galactosidase**	+	+/w/-	-	+/w/-	-	-
**β-glucuronidase**	-	+/-	-	-	-	-
**α-glucosidase**	-	-	+	+/w/-	-	-
**N-acetyl-β-glucosaminidase**	+	+	+	+/-	+	+/-
**Glycerol**	+	-	w	+/w/-	+	w/-
**D-ribose**	+	+/w	+	+	+	+
**D-galactose**	+	+/w/-	+	+	+	+
**D-glucose**	+	+	+	+	+	+
**D-fructose**	+	+/w	+	+	+	+
**D-mannose**	+	+	+	+	+	+
**Methyl-αD-glucopyranoside**	-	-	-	+/w/-	-	-
**N-acetylglucosamine**	+	+	+	+	+	+
**Esculin**	+	+/-	+	+/-	+	+/-
**D-cellobiose**	-	-	-	+/-	-	-
**D-maltose**	+	+/w/-	+	+	+	+
**D-lactose**	w	-	-	+/-	-	-
**D-melibiose**	w	-	-	-	-	-
**D-saccharose**	-	-	-	+/-	-	-
**Starch**	-	-	+	+/w	-	+/w
**Glycogen**	-	-	-	+/w/-	-	-
**Gentiobiose**	-	-	-	+/-	-	-
**D-turanose**	-	-	-	+/-	-	-
**L-fucose**	-	-	-	+/-	-	-
**Potassium 2-ketogluconate**	w	w/-	w	w/-	-	w/-
**Potassium 5-ketogluconate**	w	w/-	-	-	-	w/-
